# Imputation of Microsatellite Alleles from Dense SNP Genotypes for Parental Verification

**DOI:** 10.3389/fgene.2012.00140

**Published:** 2012-08-14

**Authors:** Matthew McClure, Tad Sonstegard, George Wiggans, Curtis P Van Tassell

**Affiliations:** ^1^Bovine Functional Genomics Lab, United States Department of Agriculture, Agriculture Research ServiceBeltsville, MD, USA; ^2^Animal Improvement Program Lab, United States Department of Agriculture, Agriculture Research ServiceBeltsville, MD, USA

**Keywords:** microsatellite imputation, parentage verification, SNP haplotype, ISAG, across breed imputation

## Abstract

Microsatellite (MS) markers have recently been used for parental verification and are still the international standard despite higher cost, error rate, and turnaround time compared with Single Nucleotide Polymorphisms (SNP)-based assays. Despite domestic and international interest from producers and research communities, no viable means currently exist to verify parentage for an individual unless all familial connections were analyzed using the same DNA marker type (MS or SNP). A simple and cost-effective method was devised to impute MS alleles from SNP haplotypes within breeds. For some MS, imputation results may allow inference across breeds. A total of 347 dairy cattle representing four dairy breeds (Brown Swiss, Guernsey, Holstein, and Jersey) were used to generate reference haplotypes. This approach has been verified (>98% accurate) for imputing the International Society of Animal Genetics recommended panel of 12 MS for cattle parentage verification across a validation set of 1,307 dairy animals. Implementation of this method will allow producers and breed associations to transition to SNP-based parentage verification utilizing MS genotypes from historical data on parents where SNP genotypes are missing. This approach may be applicable to additional cattle breeds and other species that wish to migrate from MS- to SNP-based parental verification.

## Introduction

Microsatellite markers (MS) have successfully been used for parentage verification in multiple livestock species over the past few decades. Their impact on the industry for ensuring accurate pedigree information has been immense, both for parental verification of registered animals and parental identification in multi-sire pastures (Davis and Denise, 1998; Gomez-Raya et al., 2008). Pedigree errors bias estimates of heritability, breeding values, estimates of genetic parameters, prediction of genetic gain, and depress the rate of genetic progress from selection of superior breeding animals (Israel and Weller, 2000; Senneke et al., 2004; Harlizius et al., 2011). While MS have a high polymorphic information content (PIC), allele scoring is difficult to fully automate with high accuracy because of preferential allele amplification, imperfect repeats, null alleles, and allelic dropouts (Kelly et al., 2011). Furthermore, manual scoring is prone to human error due to the complexity of patterns and data anomalies such as appearance of stutter bands (Baruch and Weller, 2008; Kelly et al., 2011). It has been estimated that MS error rate for allele scoring ranges from 1 to 5% (Baruch and Weller, 2008) to more than 30% per locus (Gagneux et al., 1997). While multiplexing can help reduce time and cost of MS panels, such as the International Society of Animal Genetics’ (ISAG) parental verification panels^1^, it can be extremely challenging to properly optimize amplification conditions to minimize error rates (Luikart et al., 2008; McClure et al., 2010).

In comparison to MS, SNP have a lower PIC due to their bi-allelic nature, but there is increasing interest from both the production agriculture and research communities to use SNP for parental verification. Advantages of SNP genotyping include minimal human interaction, lower error rates, ease of automation, and standardization between laboratories (Heaton et al., 2002; Anderson and Garza, 2006; Van Eenennaam et al., 2007; Baruch and Weller, 2008). Additionally, the genotyping cost for parental verification can be greatly reduced by using SNP, in place of MS markers. For example, Tokarska et al. (2009) estimated that using the Veracode system (Illumina Inc, San Diego, CA, USA) 50 bovine SNP can be genotyped for 1/2 the cost of 16 MS. If a set of parentage SNP were integrated into larger genotyping panels the overall price of the parental verification SNP would further decrease. This approach was employed with a set of 121 parentage SNP that were selected from a large number of candidate SNP using call-rate and allele frequencies across a large number of breeds (Heaton et al., 2002). These markers have been incorporated into all commercially available genotyping assays, including Illumina’s BovineSNP50 (Matukumalli et al., 2009) BovineHD (Illumina Inc, 2010), Bovine3K (Illumina Inc, 2011), and BovineLD (Boichard et al., 2012) and the Affymetrix BOS 1 (Affymetrix Inc, 2011).

Despite the advantages of SNP panels in cost, accuracy, and automation, transition from MS to SNP markers for parentage verification has been very slow. A major hindrance to this transition is the need for duplicate genotyping during this transition period as both parents and offspring must be genotyped with the same technology for parental verification. This requirement is a major limitation for historic animals where a DNA source does not exist due to culling, death, or change in ownership of animals. Furthermore, the additional cost of MS genotyping is difficult to justify when increasing numbers of commercial dairy cows are SNP genotyped and nearly every dairy sire is SNP genotyped. To address this issue of transition genotyping, a simple and cost-effective method is proposed to impute MS alleles from SNP haplotypes. This strategy may be implemented in any species that has dense SNP genotypes and MS alleles on a large enough subset of the population to determine phase relationships between MS alleles and SNP haplotypes.

## Materials and Methods

### Animals

Illumina Bovine High Density SNP (BovineHD; Illumina Inc, 2010) genotypes for dairy cattle (*N* = 1,301) were obtained from the industry database maintained by the Animal Improvement Programs Laboratory (AIPL) of the United States Department of Agriculture’s Agricultural Research Service (USDA-ARS)^2^. Animals from the USA represented four breeds Brown Swiss (BS, *n* = 71), Guernsey (GU, *n* = 60), Holstein (HO, *n* = 1,110), and Jersey (JE, *n* = 60; Table 1).

**Table 1:** **Animal and genotype count**.

Breed^a^	HDSNP^b^	Microsatellite^c^					
		9	10	11	12	Sire^d^	Dam^e^
BS	71	2	0	2	29	31	0
GU	60	0	5	5	6	2	0
HO	1110	6	49	118	77	395	0
JE	60	3	8	15	22	57	26
Total	1301	11	62	140	134	485	26

*^a^Breed abbreviations: BS, Brown Swiss; GU, Guernsey; HO, Holstein; JE, Jersey*.

*^b^Count of individuals with HDSNP genotypes*.

*^c^Count of individuals with genotypes for 9, 10, 11, or 12 microsatellite loci*.

*^d^Count of sires with marker genotypes from microsatellites*.

*^e^Count of dams with marker genotypes from microsatellites*.

### Genotypes

The International Society of Animal Genetics has become the *de facto* authority organization for parentage testing. The current list of MS markers was established nearly 20 years ago (Baumung et al., 2004). Genotypes for the ISAG-sanctioned 12 MS bovine panel (*BM1818*, *BM1824*, *BM2113*, *ETH3*, *ETH10*, *ETH225*, *INRA023*, *SPS115*, *TGLA53*, *TGLA122*, *TGLA126*, *TGLA227*)^3^ for offspring and their parents were obtained from the respective breed associations [US Brown Swiss Association (USBSA), USA Holstein Association (HAUSA), US Jersey Association (USJA), American Guernsey Association]. Locations of the MS on the UMD3.1 bovine genome assembly (Zimin et al., 2009) were determined by using BLAST (Altschul et al., 1990) to align reported primer sequences (Table 2).

**Table 2:** **Microsatellite information**.

Marker	Chr^a^	UMD3.1 position			Structure	Sequence	Heterozygosity^b^	MS^c^	SNP^d^	Hap^e^	Reference
		Center	Start	End							
*BM1818*	23	39,294,185	38,794,063	39,794,307	simple	(TG)_n_	79	7	32	34	Bishop et al. (1994)
*BM1824*	1	132,498,052	131,997,971	132,998,132	simple	(GT)_n_	60	5	32	19	Barendse et al. (1994)
*BM2113*	2	127,591,894	127,091,834	128,091,954	simple	(CA)_n_	69	7	14	31	Sunden et al. (1993)
*ETH10*	5	56,657,927	56,157,827	57,158,026	simple	(AC)_n_	59	8	28	40	Toldo et al. (1993)
*ETH225*	9	10,858,186	10,358,122	11,358,249	simple	(CA)_n_	71	8	24	53	Steffen et al. (1993)
*ETH3*	19	56,648,498	56,148,462	57,148,534	compound	(GT)_n_AC(GT)_6_	59	7	20	25	Toldo et al. (1993)
*INRA023*	3	33,010,922	32,510,842	33,511,002	simple	(AC)_n_		10	38	29	Vaiman et al. (1994)
*SPS115*	15	24,165,259	23,665,145	24,665,372	simple	(CA)_n_		12	10	24	Georges and Massey (1992)
*TGLA122*	21	57,640,936	57,140,875	58,140,996	compound	(AC)_n_(AT)_n_	67	15	30	51	Georges and Massey (1992)
*TGLA126*	20	22,207,036	21,706,989	22,707,083	simple	(TG)_n_	67	5	33	28	Georges and Massey (1992)
*TGLA227*	18	65,406,736	64,906,704	65,906,767	simple	(TG)_n_	81	13	34	74	Georges and Massey (1992)
*TGLA53*	16	25,789,455	25,289,387	26,289,522	compound	(TG)_6_CG(TG)_4_(TA)_n_	78	15	21	16	Georges and Massey (1992)

*^a^*Bos primigenius taurus* chromosome*.

*^b^From USDA-ARS MARC (see text footnote 2)*.

*^c^Count of unique MS alleles across all breeds*.

*^d^Count of SNP used to create haplotypes*.

*^e^Count of unique haplotypes across all breeds*.

BovineHD SNP genotypes for markers within 500 kb of each MS (average 301 SNP) were obtained from USDA-ARS AIPL. These genotypes represent animals from the HapMap population; animals genotyped in research projects at the USDA-ARS Bovine Functional Genomics Laboratory (see text footnote 2); and animals with data exchanged as part of Cooperative Dairy DNA Repository Steering Committee’s collaborations with the Canadian Dairy Network, DairyCo of the United Kingdom, and ANAFI of Italy. SNP data was in AB format from GenomeStudio (Illumina).

All genotype data was loaded into SVS7 [SNP and Variation Suite v7.5; (Golden Helix, Bozeman, MT, USA]. Minor allele frequencies (MAF) at all loci were required to exceed 5% across breeds.

### Haplotype identification

The BEAGLE program (Browning and Browning, 2007) was used to phase the genotypes, as this software was one of the few that could handle both bi- and multi-allelic data. SNP loci used for phasing and the DNA base associated with “A” and “B” alleles are listed in Table S1 in Supplementary Material. Genotypic data for 347 animals (33 BS, 16 GU, 250 HO, 48 JE, Table 1) that had both MS and flanking SNP data (the “combined” data set) was exported from SVS7, and each breed was independently phased by BEAGLE using 1,000 iterations.

Haplotypes were initially determined using 10 SNP and the MS genotype centered on a MS. Haplotypes of increasing sizes were analyzed by counting the number of times each MS allele was phased with each SNP haplotype (MS-haplotype). The extension of a haplotype was ended when a haplotype size was identified that sufficiently fit the following conditions:

1. Minimize the number of MS alleles associated with a haplotype within a breed (ideally, each haplotype associated with a single MS allele),
2. Minimize the number of singular MS-haplotype association counts,
3. Minimize the number of haplotypes with multiple MS alleles associated with a single haplotype,
4. Minimize the total number of SNP needed to impute MS alleles.

Table 3 illustrates the *BM1824* allele counts per haplotype for the 32 SNP haplotype that satisfy the above criteria. The resulting counts were condensed into an MS imputation table (Table S2 in Supplementary Material) showing the fraction of MS alleles observed with each haplotype by breed.

**Table 3:** **Example of allele counts per SNP haplotype for BM1824 that fits the haplotype identification criteria**.

Haplotype^a^	Brown Swiss					Guernsey					Holstein					Jersey				
	178^b^	180	182	188	190	178	180	182	188	190	178	180	182	188	190	178	180	182	188	190
BABBAABBAAAABBBAABABABBBABBBAAAB	26					5					32					1				
BAABAABBAAAABBBAABABABBBABBBAAAB											32									
BBBBAABBAAAABBBAABABABBBABBBAAAB											21									
BABBAABBAAAABBBAABABABBBAABBBBAB	4																			
BABBAABBAAAABBBAABABABBBABBBAABA	3																			
BAABBAABBAAABBBAABABABABABBBAAAB						2														
BAABABABBAAABBAABAABABBBABAAAABA												65		1						
BAABABABBAAABBAABAABABBBABBBAAAB												50								
AABBAABBBAAABBAABAABABBBABBBAAAB																	58	2		
AAAABAAABBABBAAABAABABABABAAAAAB		3					14													
AAAABAAABBABBAAABAABABABABAAAABA			8					8					69				1	3		
AAAABAAABBABBAAABAABABABABAAAAAA													14							
BAABBAABBBABBBAABBABABABABABAAAA													1							
AAAABAAABBABBAAABAABABABABABAAAA																		3		
BABBAABBAABAABBBBBBABAAABABBBBAB								1												
BAAABABBAABAABBBBBBABAAABABBBBAB														113						
BAABAABBAABAABBBBBBABAAABABBBBAB								1						71						
AABBAABBAABAABBBBBBABAAABABBBBAB				3															4	
AAAABABBAABAABBBBBBABAAABABBBBAB				19					1					8	9				3	11

*^a^Haplotypes SNP listed in Table S2 in Supplementary Material*.

*^b^BM1824 alleles*.

### Within breed imputation

Genotypes for the SNP from haplotypes identified above (Table S2 in Supplementary Material) were independently phased by breed (BS, GU, HO, and JE) for 1,301 animals in BEAGLE. The 347 animals used to generate the haplotypes were a subset of the 1,301. The resulting SNP haplotypes were then compared to those determined using the combined SNP and MS genotypes and listed in the breed specific imputation table (Table S2 in Supplementary Material). For novel SNP haplotypes not observed in the combined data, no MS allele was inferred. As a result of these non-predicted genotypes, an animal could have two, one, or no MS alleles imputed. Only MS-haplotype information within breeds was used to impute MS alleles.

### Across breed imputation

When multiple MS alleles were associated with a haplotype within a breed the less frequent allele was rarely observed over 2 times. While multiple MS alleles could be associated with a haplotype because of higher mutation rates of MS compared with SNP (4.5 × 10^−5^ vs. 1 × 10^−5^; Ellegren, 1995; Falconer and Mackay, 1996), there is a much higher chance that this discrepancy resulted from MS genotyping errors. To better account for low-frequency improper assignments of MS alleles with SNP haplotypes, an alternative MS imputation was devised where a MS-haplotype association had to be observed a minimum four times for that association to be considered for MS imputation.

Analysis of the imputation table (Table S2 in Supplementary Material) also revealed that MS-haplotype combinations often held true across breeds. To assess if MS-haplotype combinations were present before modern breed formations, and have been preserved across breeds, MS-haplotype information from other breeds was used to impute alleles if the haplotype was not observed in the breed during the initial haplotype identification.

### Validation

Imputed and breed association reported MS genotypes were compared as follows: (1) parentage verification using only reported MS, (2) parentage verification using individual imputed MS and observed parental MS genotypes, and (3) concordance between the reported and imputed MS genotype. When multiple MS alleles were associated with a SNP haplotype, all associated MS alleles were considered as possible alleles in the analysis. Parentage verification was performed by locus and failed if two putative MS allele could not have been inherited from either parent.

## Results

For all MS loci tested, the 424 SNP-based haplotypes identified from 316 SNP (Tables S1 and S2 in Supplementary Material) effectively identified MS alleles for the analyzed breeds. When using the within breed imputation method 358 haplotypes were associated with a single MS allele, 50 of these unique associations were observed in at least two breeds. A total of 165 of these breed specific associations were seen only in Holstein, possibly because of the larger data set for that breed. While 358 haplotypes were associated with one MS allele, 11 were associated with a single MS allele within a breed and multiple alleles across breeds, with the remaining 55 associated with multiple MS alleles in at least one breed. Considering the numbers of genotypes in the combined data set, these counts were 5,945, 260, and 1,487, respectively.

With the across breed imputation method 384 haplotypes were associated with a single MS allele, 65 of these being observed in at least two breeds. For the remaining haplotypes: 21 were associated with single MS allele within a breed and multiple alleles across breeds, and 19 were associated with multiple MS alleles in at least one breed. Considering the numbers of genotypes in the combined data set, these counts were 6,366, 498, and 828, respectively.

While using only within breed imputation resulted in a slightly higher concordance rate between an individual’s reported and imputed MS allele (99.4 vs. 98.0%) 334 more genotypes were imputed using the across breed imputation method across the 1,301 individuals (Table 4 and Table S3 in Supplementary Material). On a per locus basis, parentage verification rates were 99.8% for MS reported alleles, 99.4% for within breed imputed MS alleles, and 99.2% for across breed imputed alleles. Statistically, these rates are not different (*P* > 0.13; Table 5).

**Table 4:** **Percent of 1,301 individuals with haplotypes imputed to microsatellite (MS) alleles**.

MS	Alleles^a^	0 Allele^b^		1 Allele		2 Allele	
		Within^c^	Across^d^	Within	Across	Within	Across
*BM1818*	7	0.020	0.012	0.980	0.988	0.834	0.858
*BM1824*	5	0.001	0.001	0.999	0.999	0.983	0.988
*BM2113*	7	0.004	0.001	0.996	0.999	0.960	0.985
*ETH10*	8	0.010	0.003	0.99	0.997	0.940	0.949
*ETH225*	8	0.037	0.018	0.963	0.982	0.902	0.932
*ETH3*	7	0.005	0.005	0.995	0.995	0.947	0.957
*INRA023*	10	0.012	0.002	0.988	0.998	0.947	0.961
*SPS115*	7	0.006	–	0.994	1.000	0.961	0.988
*TGLA122*	15	0.044	0.017	0.956	0.982	0.891	0.904
*TGLA126*	5	0.015	0.009	0.985	0.991	0.944	0.960
*TGLA227*	13	0.033	0.028	0.967	0.972	0.889	0.892
*TGLA53*	15	0.002	–	0.998	1.000	0.954	0.971
Average	8.9	0.016	0.008	0.984	0.992	0.929	0.945

*^a^Number of unique MS alleles*.

*^b^0 = MS alleles were not imputed from either haplotype, 1 = MS allele imputed from only 1 haplotype, 2 = MS alleles imputed from both haplotypes*.

*^c^MS imputation performed only using within breed haplotype data*.

*^d^MS imputation performed using across and with breed haplotype data*.

**Table 5:** **Microsatellite (MS) validations**.

Microsatellite	Concordance^a^				Parentage verification^b^						
	Within^g^	Across^h^	*N*^i^	Reported MS^c^		Imputed MS^d^								
					*N*	Overall			Not reported^e^			Reported^f^		
						Within	Across	*N*	Within	Across	*N*	Within	Across	*N*
*BM1818*	0.993	0.977	133	1.000	37	0.972	0.979	144	0.991	0.991	107	0.921	0.947	38
*BM1824*	1.000	0.994	337	0.995	189	1.000	1.000	475	1.000	1.000	289	1.000	1.000	188
*BM2113*	0.991	0.980	344	1.000	195	0.998	0.998	478	1.000	1.000	284	0.995	0.995	194
*ETH10*	1.000	0.988	343	1.000	194	0.996	0.987	479	0.997	0.986	285	0.995	0.990	195
*ETH225*	0.988	0.955	325	1.000	154	0.991	0.979	470	0.989	0.975	283	0.995	0.984	187
*ETH3*	0.997	0.991	340	1.000	149	1.000	1.000	479	1.000	1.000	288	1.000	1.000	192
*INRA023*	0.991	0.988	343	0.990	194	1.000	1.000	479	1.000	1.000	285	1.000	1.000	194
*SPS115*	0.979	0.954	340	1.000	195	0.996	0.994	479	0.997	1.000	284	0.995	0.985	196
*TGLA122*	0.997	0.959	319	1.000	195	0.996	0.979	471	0.993	0.986	282	1.000	0.958	189
*TGLA126*	0.997	0.988	345	1.000	194	1.000	1.000	478	1.000	1.000	284	1.000	1.000	194
*TGLA227*	1.000	0.979	337	1.000	189	0.993	0.989	475	0.990	0.990	286	1.000	0.989	190
*TGLA53*	1.000	0.970	297	0.987	153	0.991	0.993	431	1.000	1.000	279	0.974	0.980	152
Average	0.994	0.977	317	0.998	170	0.994	0.992	445	0.996	0.994	270	0.989	0.986	176

*^a^Percent of imputed MS allele(s) match the reported alleles*.

*^b^Percent of individuals per locus that pass parentage verification*.

*^c^Parentage verification using only breed reported MS alleles on the individual and its parents*.

*^d^Parentage verification using imputed MS alleles on the individual and breed reported parental MS alleles*.

*^e^Individuals did not have a breed reported MS allele*.

*^f^Individuals that had a breed reported MS allele*.

*^g^MS imputation performed only using within breed haplotype data*.

*^h^MS imputation performed using across and with breed haplotype data*.

*^i^Count of individual*.

## Discussion

Techniques must be developed to bridge traditional and new technologies in order to allow producers to benefit from cheaper parentage assays while maintaining compatibility with previous historic data; thus avoiding additional costs of re-genotyping the parental generation. The method developed here represents an effective and inexpensive way for the livestock genetics community to parentally verify an individual when separate genotyping platforms have been used across the generations. There are currently a large number of active North American dairy cattle that could use MS imputation for parentage verification. The HAUSA currently uses both SNP- and MS-based methods for parental verification, and from 3/2011-3/2012 845 bulls and 7,468 dams (out of a total of 8,643 and 236,406 respectively) were verified using MS only (Tom Lawlor-HAUSA, Personal communication 3/19/2012). In a similar manner the USJA has 1,918 dams in the breeding herd that only have MS genotypes (Erick Metzger-USJA, Personal communication 3/27/2012). Starting in 2012 the USJA has begun using only SNP for parentage verification. MS imputation would enable USJA, and others (Dave Kindall-USBSA, Personal communication 4/4/2012) to eliminate the need to re-test these dams with SNP. For researchers this method will allow for the merging of historic and current data even though they are derived from different types of markers.

**Figure 1:**
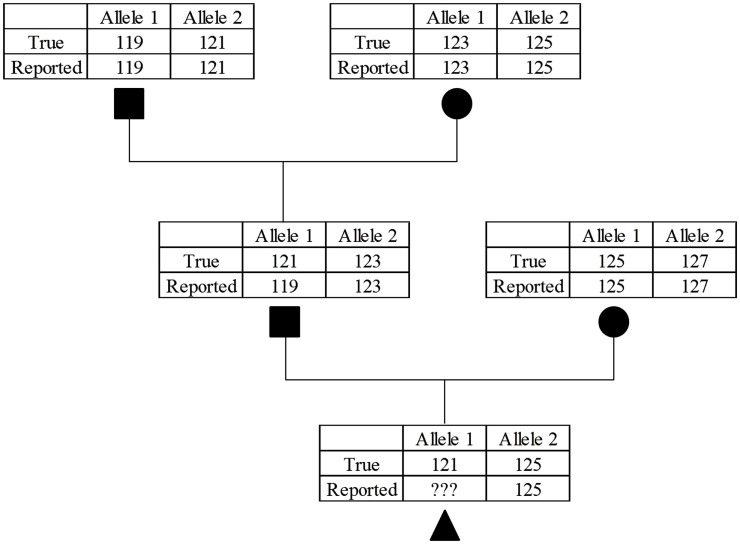
**Example of the compounding effect of MS allele miscalls**. While the sire in the second generation was misgenotyped for the parental allele it was not caught because the error still follows Mendelian inheritance patterns. This will cause a problem in the third generation because if the parental 121 allele is called correctly it will fail the parentage verification, if incorrectly called again as a 119 allele this error will further propagate in future generations.

Scant literature exists that report MS genotyping error rates, but Bonin et al. (2004) estimated an error rate of 0.8% per allele in bears. These “errors often go undetected because they are generally unobtrusive” (Bonin et al., 2004). A random set of genotyping errors were not present in this study, because the genotypes used were those confirming parentage. If a majority of marker genotypes provide support for a parent-progeny relationship, then the genotypic data would likely be revisited and potentially revised. This problem can be envisioned by considering a case where a bull had an improperly scored allele for a MS genotype which did not violate Mendelian inheritance (Figure 1). Depending on the genotype of the bull’s mate, this situation may result in forcing the genotype to be incorrect to avoid non-inheritance of MS alleles. This situation could be problematic in the vignette described, because the bull’s genotypes would precede his offspring by a considerable time, and the sire’s genotype would be considered correct (and quite possibly no DNA available for retesting). A practical consideration is that it is likely that the same chemistry conditions that led to the original allelic dropout or false alleles would occur again. Regardless of the cause, these genotyping errors would result in incorrect haplotype structures.

## Conclusion

This research represents the first time an accurate method has been developed to that can impute multi-allelic genotypes from bi-allelic data. Our results will have an immediate impact for livestock associations wishing to transition from MS- to SNP-based parentage verification. Additionally, the same methods can be used to impute MS alleles for studies that desire to combine data sets that include both MS and SNP genotypes. MS-haplotype combinations that hold true across multiple phylogenetically unique cattle breeds (Decker et al., 2009; Table S2 in Supplementary Material) may represent historic MS-haplotypes. In theory, these MS-haplotypes could be used to accurately impute MS alleles in other *Bos primigenius taurus* breeds using the imputation (Table S2 in Supplementary Material) reported here.

For commercial application of this method, it is recommended that the SNP listed in Table S1 in Supplementary Material be used as a standard set thus allowing for easy imputation and standardization across labs and platforms. While these SNP are all present on the BovineHD assay, it is possible that some SNP will not be compatible with other commercial platforms. For haplotypes that are associated with >1 MS alleles, the percent of times each association is seen in the test population (Table S2 in Supplementary Material) could be incorporated in exclusion probabilities for parentage identification in multi-sire mating populations, in a manner similar to allele frequencies. A built in benefit of our method is that while the selected SNP provide excellent performance a reduced set of SNP could be used for imputation, although this could result a lower resolving power and accuracy. As more animals and breeds with accurate MS and SNP genotypes are collected a reanalysis with all of the data will result in increased resolving power, identification of rare MS-haplotype combinations, and potentially identification of combinations that can be used across breeds not tested in this study.

While MS are currently the international standard parentage verification for exported semen, this work represents a tool to quickly migrate toward *SNP-based verification in one generation*. Finally, these imputation methods can be implemented in any species with available high density SNP genotypes flanking MS allele genotypes.

## Conflict of Interest Statement

The authors declare that the research was conducted in the absence of any commercial or financial relationships that could be construed as a potential conflict of interest.

## Supplementary Material

The Supplementary Material for this article can be found online at http://www.frontiersin.org/Livestock_Genomics/10.3389/fgene.2012.00140/abstract

## Footnotes

^1^http://bit.ly/ISAG_pv

^2^http://www.marc.usda.gov/genome/cattle/cattle.html

^3^http://www.isag.us/Docs/CattleMMPTest_CT.pdf
